# Whooping Crane Chick Survival in the Reintroduced Eastern Migratory Population

**DOI:** 10.1002/ece3.71284

**Published:** 2025-04-25

**Authors:** Hillary L. Thompson, Andrew J. Caven, Stephanie M. Schmidt, Bianca R. F. Sicich, Alexis J. Sarrol, Eva K. Szyszkoski, Nicole M. Gordon

**Affiliations:** ^1^ International Crane Foundation Baraboo Wisconsin USA; ^2^ Louisiana Department of Wildlife and Fisheries Gueydan Louisiana USA

**Keywords:** *Grus americana*, nesting, parental experience, recruitment, reintroduced population, reproduction, survival, whooping crane, Wisconsin

## Abstract

The reintroduced Eastern Migratory Population (EMP) of Whooping Cranes ( *Grus americana* ) has exhibited appropriate breeding behavior, including pair formation, territory defense, nest building, and fertile egg production. However, recruitment has been lower than what is needed for a self‐sustaining population due to high chick mortality. During 2006–2023, 194 chicks hatched in the EMP, with only 36 surviving to fledging. For the population to succeed without continued releases of captive‐reared individuals, we must develop management strategies that increase recruitment to a level above mortality rates. We examined apparent weekly survival data of wild‐hatched Whooping Crane chicks collected via aerial and ground surveys using radio telemetry from 2006 to 2023. In this study, we explored relationships between chick survival and a host of potentially impactful predictor variables including parental experience, parental life history, habitat, ecoregion, weather, and climate, as well as nest and clutch characteristics using Cox Proportional Hazard Regression Models. Our results indicate that a chick without a sibling has an increased probability of survival. Survival probability also increased with collective parental experience and warm days (> 32°C) during the first 4 weeks after hatch. Our data indicate that parental experience is a reliable predictor of recruitment. Adult survival may therefore be indirectly linked with low chick survival as experienced adults are too often lost from this population. Additionally, our results suggest that efforts to collect a single egg from two‐egg nests may improve weekly survival of Whooping Crane chicks.

## Graphical abstract

The reintroduced Eastern Migratory Population (EMP) of Whooping Cranes ( *Grus americana* ) has experienced recruitment rates lower than what is needed for a self‐sustaining population due to high colt mortality. We examined weekly survival data of wild‐hatched Whooping Crane colts from 2006 to 2023 and explored relationships with predictor variables including parental experience, habitat, ecoregion, weather, and nest or clutch characteristics. Whether a colt had a sibling, if parents had more experience raising young, and warm days during the first 4 weeks after hatch were reliable predictors of crane colt survival.

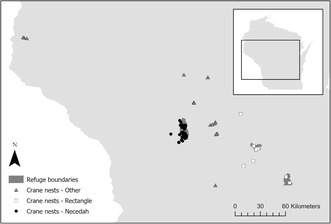

Whooping Cranes ( *Grus americana* ) are an endangered species of bird with three distinct populations in North America: one remnant migratory population and two reintroduced populations. To downlist the species from endangered to threatened, the International Recovery Plan outlines population goals for the remnant migratory population as well as both reintroduced populations (CWS and USFWS 2007). One of the current reintroductions is the Eastern Migratory Population (EMP) in the eastern United States, which started with the first cohort of captive‐reared juveniles released in 2001. The recovery criteria for reintroduced populations specify that each population should be self‐sustaining with at least 100 individuals and 25 breeding pairs (CWS and USFWS 2007). As of 2024, approximately 75 Whooping Cranes survive in the EMP with 22 breeding pairs, and natural recruitment rates (wild‐hatched chicks surviving to reproduce themselves) remain lower than mortality rates (ICF 2024a; Thompson et al. 2022b).

Poor nest success and high chick mortality both have contributed to low recruitment rates; however, most studies have focused on factors influencing nest success (Barzen et al. 2018; Converse et al. 2013; Converse et al. 2019; King et al. 2013b; Urbanek et al. 2010). Whooping Cranes are a long‐lived species that can live over 20 years in the wild and typically start breeding at 3–5 years of age (Thompson et al. 2021). In the EMP, crane pairs tend to be monogamous within and across breeding seasons and have only 1–2 clutches or nesting attempts per season (very rarely 3 clutches). One cause of nest failure in the EMP has been disturbance by avian‐feeding blackflies (*Simuliidae* spp.) at the core breeding area, Necedah National Wildlife Refuge (NWR) in Wisconsin (Converse et al. 2013; Converse et al. 2019; Urbanek et al. 2010). The effects of blackflies have been partially mitigated by removing eggs from first nests susceptible to abandonment after blackfly emergence, thus encouraging pairs to re‐nest once the seasonal blackfly population has reduced (Adler et al. 2019; Jaworski 2016; Thompson et al. 2022b). Additionally, recent reintroduction efforts have occurred in eastern Wisconsin, in areas with fewer blackflies present (Adler et al. 2019; Thompson et al. 2022b; Van Schmidt et al. 2014). These management techniques have resulted in higher numbers of chicks hatching in the wild. However, even with higher rates of nest success, chick survival and fledging rates remain low (Barzen et al. 2018; McLean 2019; Thompson et al. 2022b).

Few studies have focused on chick survival rates or cause‐specific mortality of Whooping Cranes in the EMP (King et al. 2013a; McLean 2019; Stewart 2020; Thompson et al. 2022b); however, studies of semi‐precocial and precocial chicks have revealed relationships between juvenile survival and parental experience, habitat characteristics, and demography. Greater parental age and experience have been attributed to increased juvenile survival (Blomqvist et al. 1997, Dreitz 2009). Parental care of precocial chicks includes brooding, defense, and provisioning. Adults with greater experience may have more effective parenting behaviors and better access to quality sites (Blomqvist et al. 1997). Greater juvenile survival also has been associated with nest‐site and posthatch habitat selection that reduces exposure to predators and improves food availability (Blomqvist et al. 1997, Dreitz 2009, Fox et al. 2019). For Whooping Cranes, this could include a selection for emergent herbaceous wetlands (i.e., nesting habitat) and open uplands (i.e., foraging habitat), and an avoidance of forests or densely vegetated areas which can conceal predators (Paton 1994; Van Schmidt et al. 2014). Additionally, juvenile survival has been found to increase with the individual's age (Fox et al. 2019; Maness and Anderson 2013; Schmidt 2022; Severud et al. 2022), presumably due to greater mobility, size, and predator avoidance skills gained across the course of development (Jones et al. 2017; Schmidt 2022). Chick age and clutch size also are reported to have an impact on crane chick survival, with greater survival for 1‐egg clutches and older chicks in 2‐egg clutches due to a competitive advantage over or lack of resource contestation and aggression from a sibling (Bergeson et al. 2001). Sibling fates also have been positively linked, suggesting the mortality of one crane chick increases the risk of mortality for the second (USFWS 2014). Here we use sibling presence to assess these factors since there were no 3‐egg clutches and were not able to differentiate between an older or younger chick in a 2‐egg clutch in which both eggs hatched. Finally, single eggs have been removed from a subset of 2‐egg clutches in the EMP to potentially increase the survival of the remaining chick and to increase the number of eggs for captive‐rearing and release into the reintroduced populations (ICF 2024b).

The objective of this study was to determine factors influencing wild‐hatched Whooping Crane chick survival from 2006 to 2023 in the EMP. Here we investigate effects on wild‐hatched chick survival rates of a variety of covariates including nest and chick characteristics (nest order or the nest attempt by a pair during a single season (e.g., first, second, third), clutch size, hatch date, sibling presence, sex, egg management), parental experience (male and female parent ages and summed ages, each parent's years of nesting experience, the pair's summed years of nesting experience, and each parent's previous hatch/fledge success), parental rearing history (captive vs. wild hatch, parent‐rearing vs. costume rearing), habitat and region (nest location, region, ecoregion, adults' winter location the previous season), and weather and climate (precipitation, soil moisture, solar irradiance) variables. By evaluating factors affecting Whooping Crane chick survival, our analysis will inform the development of management strategies to increase recruitment in the EMP. Furthermore, these methods and results could be used to inform reintroduction or conservation efforts of other threatened or endangered species, especially those with captive‐rearing or nest management as a part of their recovery strategies.

## 1 Study Areas

**FIGURE 1:**
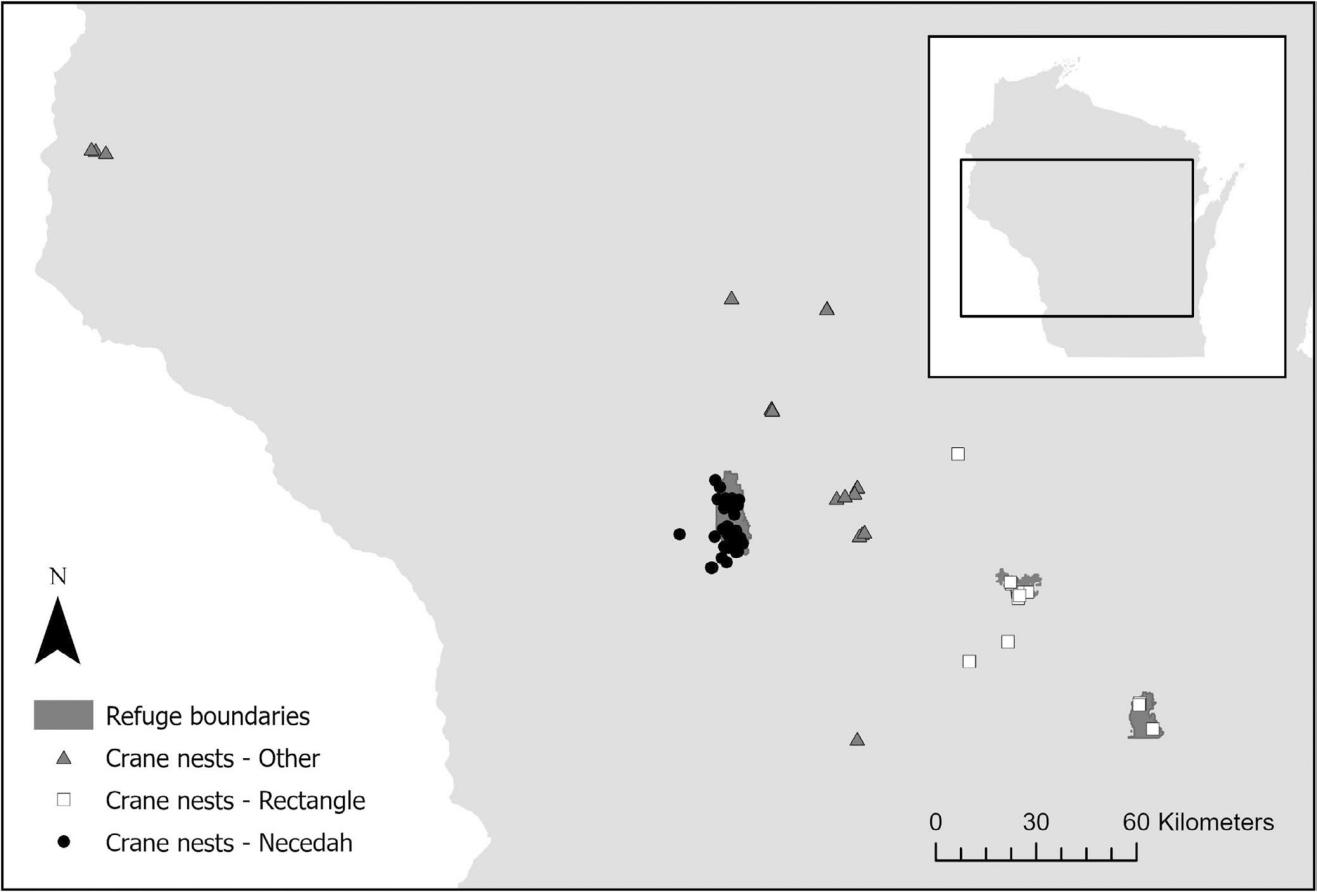
Map of all Whooping Crane nests in Wisconsin, USA, to date (2006–2023 nesting seasons, inclusive) in the reintroduced Eastern Migratory Population that have successfully hatched a chick. The three refuges on the map from west to east are Necedah National Wildlife Refuge (NWR), White River Marsh State Wildlife Area (SWA), and Horicon NWR. Whooping Cranes nested at or near release areas at Necedah NWR, and an area known as the rectangle in eastern Wisconsin, which includes White River Marsh SWA and Horicon NWR, as well as areas outside of the release areas (other).

All wild‐hatched Whooping Crane chicks in the EMP from 2006 to 2023 hatched within Adams, Dodge, Green Lake, Juneau, Marathon, Marquette, Monroe, Portage, Sauk, St. Croix, and Wood Counties, Wisconsin (Figure 1). The majority (60.3%) of successful hatches have occurred at Necedah NWR, from nests in managed wetland complexes dominated by emergent vegetation, including sedges (*Carex* spp.; Van Schmidt et al. 2014; Strobel and Giorgi 2017). Necedah NWR and the surrounding areas in central Wisconsin are within the footprint of the historic Glacial Lake Wisconsin, an area characterized by sandy soils (WDNR 2015b). Nest sites in eastern Wisconsin, in an area known as the Eastern Rectangle (Thompson et al. 2022b; Van Schmidt et al. 2014), were concentrated at White River Marsh State Wildlife Area (SWA), Grand River Marsh SWA, and Horicon NWR. The wetlands used by cranes in the Eastern Rectangle in the Southeast Glacial Plains ecological landscape are dominated by sedges and cattails (*Carex* and *Typha* spp.; WDNR 2015b) and are surrounded by an agricultural landscape. Overall, a majority (77.7%) of chicks were hatched on protected lands, including Necedah NWR, Meadow Valley SWA, George W. Mead SWA, Colburn SWA, McMillan Marsh SWA, White River Marsh SWA, Grand River Marsh SWA, and Horicon NWR. The remaining chicks hatched in wetlands on private lands, many of which were on smaller wetlands than those at protected areas and were surrounded by agricultural areas. Additionally, some wetlands on private properties were used as reservoirs for cranberry farming operations. Generally, nesting areas in wetlands on private properties were comprised of similar vegetation communities as nearby protected wetlands. However, they may have been closer to development and experienced greater human disturbance.

## 2 Methods

### 2.1 Whooping Crane Reintroduction and Monitoring

From 2001 to 2023, 36 Whooping Cranes fledged from nests in the EMP, while 311 Whooping Cranes were released after being raised in captivity by costumed handlers (costume‐rearing) or captive adult Whooping Cranes (parent‐rearing) (ICF 2024b; Thompson et al. 2022b; Urbanek and Bookhout 1992). The captive‐reared Whooping Cranes were either trained to follow an ultralight aircraft or wild adult Whooping Cranes on their first southward migration from their release site in Wisconsin to a wintering site (Hartup 2019; Thompson et al. 2022b).

Prior to the release of captive Whooping Cranes or fledging of the majority of wild‐hatch Whooping Cranes, each bird was fitted with a leg band‐mounted VHF radio transmitter (Very High Frequency or VHF; Advanced Telemetry Systems, Isanti, MN; Urbanek 2018), a unique color combination of leg bands, a United States Geological Survey (USGS) band with a unique number, and occasionally a GPS tracking device, either satellite Platform Terminal Transmitters (PTTs; Microwave Telemetry, Columbia, MD) or a cellular‐based Global System for Mobile Communications (GSM; Microwave Telemetry, Columbia, MD). During the banding of captive‐reared or wild‐hatched Whooping Cranes, blood was drawn to determine the sex of the chick using genetic techniques (Duan and Fuerst 2001; Griffiths et al. 1998).

Whooping Cranes in the EMP were regularly monitored through visual observations (on the ground and aerial), radio‐tracking (VHF: 7‐element yagi antenna; Cushcraft Corporation, Manchester, NH), satellite telemetry, and reported sightings from the public. During the breeding season, pairs were monitored daily to weekly, and nests were located when pairs exhibited breeding behaviors or during aerial monitoring flights. Nests continued to be monitored throughout the incubation period, and the hatch date of chicks was determined either by the first visual or trail camera (Trophy Cam model 119,466, Bushnell, Overland Park, KS, USA; HyperFire HC600, Reconyx, Holmen, WI, USA) observation of a chick with parents (Jaworski 2016; McKinney 2014). Two eggs from the same nest generally hatched within 1–2 days of each other, and we were not able to distinguish a difference between the first‐ and second‐hatched chicks from a single clutch. During the chick‐rearing season, breeding pairs known to have chicks were monitored closely via aerial and ground surveys, and pairs were observed until it could be confirmed if their chick or chicks were still alive. If a breeding pair or family group was not found during a survey, another survey was conducted during the week so we could confirm if a chick was alive or not during each week of the prefledge period. For all chicks in this study, we confirmed weekly survival until a pair was consistently no longer seen with their chick or the carcass of the chick was recovered.

### 2.2 Data Collection

#### 2.2.1 Nests and Chicks

During regular observations at located nests from 2005 to 2023 (visual and camera), we recorded clutch size, defined as the number of eggs on the nest prior to pulling an egg for management purposes. Prior to nest monitoring, we also recorded the nest order, defined as the first, second, or third nest of the season for an individual pair. Hatch date was recorded when chicks were estimated or known to have hatched from visual or camera observations. Following hatch, each chick from a nest was given an identification number. Each chick was assessed weekly for 12 weeks after initial detection, which covers the period in which cranes fledge or learn to fly. Prior to separating from their parents, chicks were captured for banding and sex was determined at this time. Sex was undetermined for chicks who were not captured or whose carcasses were not recovered.

#### 2.2.2 Parental Experience

We used long‐term visual, camera, and radio monitoring records from prior years to document female and male parents' age and years of nesting experience. Nesting experience may be indicative of a parent's ability to successfully hatch and fledge young (Blomqvist et al. 1997, Dreitz 2009) and included the year of a female/male's first nest, the number of years the pair have nested inclusive of other partners (first year nesting = 1), and if they have successfully hatched and/or fledged a chick in previous years. We collected female and male parents' variables both individually and summed to measure the experience of the pair.

#### 2.2.3 Parental Rearing History

Female and male parents' rearing and release history were recorded to capture the impact of rearing strategies on their ability to successfully hatch and fledge chicks in the wild. Rearing methods included costume‐reared, parent‐reared, and wild‐hatched (i.e., chicks hatched on nests in the wild). We also documented the release strategy used for the female and male parents and included ultralight, Direct Autumn Release, parent‐reared with a hard release, and wild‐hatched (i.e., no release strategy used). Ultralight‐released birds were reared using costume‐rearing methods and followed an ultralight aircraft to guide them south on their first migration to their wintering site, where they were soft‐released. Alternatively, Direct Autumn Release birds also were costume‐reared in captivity but were soft‐released into an acclimation pen on a refuge or state wildlife area before following adult cranes to their wintering location. Finally, parent‐reared birds were reared in captivity and hard‐released near wild adult Whooping Cranes to follow them on their first migration.

#### 2.2.4 Habitat and Region

We recorded each nest location, including the region defined as Necedah NWR, eastern rectangle (i.e., Horicon NWR, White River Marsh SWA, and Grand River Marsh SWA), and others (Figure 1). The ecoregion of the nest site was categorized using definitions from the Wisconsin Department of Natural Resources Ecological Landscapes of Wisconsin (WDNR 2015b). We also documented if the nest was located within a protected area and estimated habitat characteristics at the nest location using multiple sources of remotely sensed data. We used the Wisconsin Wetlands Inventory to identify wetland type and size of wetland at the nest site (WDNR 2015a). We grouped wetland classes into three broad categories: open/emergent, shrubby, and forested wetlands. In ArcGIS 10.6.1, we used the Spatial Join tool to identify wetland polygon characteristics at each nest site that hatched a chick (ESRI 2011). To approximate habitat types available to flightless chicks, we used the Buffer Tool in ArcGIS to create a 4.58 km^2^ buffer around each nest location, similar to average home range sizes used by breeding Whooping Cranes in the EMP (Barzen et al. 2019; ESRI 2011). Using the Tabulate Intersection tool, we calculated the percentage of each buffer categorized as wetland by the Wisconsin Wetlands Inventory (ESRI 2011; WDNR 2015a). Forests may provide cover for potential predators of flightless chicks or eggs in nests (Paton 1994), so we included a variable to indicate if forested habitat was present around the nest site using the WiscLand 2.0 dataset (WDNR—UWM 2016). Finally, using prior observations of the parents, we recorded site characteristics of their wintering location, including the state and region, as defined by Thompson (2018; north region includes Indiana, Illinois, Kentucky; central region includes Tennessee, Alabama; southern region includes Georgia, Florida, Louisiana).

#### 2.2.5 Weather and Climate

Climate and weather data were collected from the nearest weather station to the nest location where daily data were available, identified through the Midwestern Regional Climate Center (cli‐MATE 2024). Nesting season precipitation and temperature (i.e., freezing temperatures < 0°C, Butler et al. 2017; extreme heat days > 36°C, Woolley et al. 2022) have been identified as drivers of population size and reproductive potential in cranes. To measure the effects of nesting season weather patterns on Whooping Crane chick survival, we documented total and cumulative precipitation (centimeters), maximum, minimum, and average temperatures, and weeks with temperatures > 32°C and < 0°C. Heat stress in adult Whooping Cranes in Wisconsin occurs at temperatures exceeding 36°C (Fitzpatrick et al. 2015). However, conservative estimates must be considered when measuring chick heat stress in northern climes, compared to adult cranes; thus, we chose to evaluate temperatures over 32°C rather than 36°C. We also considered weather and climate covariates within further divided 4–5 week segments to represent early, mid‐, and late prefledging chick developmental stages. These developmental stages have been characterized as having differential mortality rates (e.g., King et al. 2013a). Additionally, using larger and relatively equally spaced periods helped limit weekly stochasticity for analytical purposes. We also documented indicators of climate change, known to be drivers of Whooping Crane population vital rates (Butler et al. 2017). Variables included yearly mean sunspot numbers (WDC‐SILSO 2015), a measure of solar activity further influencing temperature, weather patterns, plant growth, surface hydrology, and animal activity. However, the mechanisms behind the impact of sunspots on Whooping Cranes remain unknown and may be more pronounced at the more northern breeding areas of the Aransas‐Wood Buffalo Population of Whooping Cranes (Butler et al. 2017). We also included a measure of soil moisture (PDSI) in June to differentiate relative dry and wet conditions (NOAA 2023).

### 2.3 Data Analysis

All statistical analyses were conducted in the open‐source statistical software program R version 4.3.2 (R Core Team 2023). We assessed predictors of apparent chick survival across 12 periods over 13 survey weeks after hatching with Cox Proportional Hazard Regression Models (Cox 1972; Therneau and Grambsch 2000) using the “survival” package (Therneau 2023). We conducted a two‐stage modeling process. Multitiered modeling procedures reduce the number of competing multivariate models in the final stage of analysis to the most informative set of predictors, which can improve the clarity and performance of model selection approaches (Burnham and Anderson 2002; Caven et al. 2022; Franklin et al. 2000; Ranglack et al. 2017). First, we ran 53 bivariate models with the weekly probability of chick survival as the outcome variable and individual variables, including some multilevel factors, as predictors. These models were compared to respective null models with the same data using a Likelihood Ratio Test (Buse 1982) using the “lmtest” package (Zeileis and Hothorn 2002). This approach allowed us to maximize our available data when evaluating bivariate relationships. We also calculated a conservative measure of explained variation (MEV) for each model following Royston (2006) using the “survMisc” package (Dardis 2022). Values for this metric tend to be lower than traditional *R* ^2^ measures but can be interpreted similarly as a relative estimate of model fit to the data.

We advanced models that were significantly better than the null to a second tier of analysis where we also created a priori multivariate models with uncorrelated combinations of those predictor variables. No two variables with a Pearson correlation coefficient > 0.6 were included in the same model (Dormann et al. 2013). All predictor variables were categorized into broader themes including *nest and chick elements*, *parental experience*, *parental rearing history*, *habitat and region*, and *weather and climate covariates*, which allowed us to elucidate larger patterns in the data. All multivariate models included at least one variable from each theme (hereafter, global models) that advanced to the second tier of analysis. We ranked second‐tier models with Akaike Information Criterion corrected for small sample sizes (AICc) using the “MuMIn” package (Barton 2020; Burnham et al. 2011; Burnham and Anderson 2002). We presented bivariate results from tier one as well as outcomes from all models within an AICc Delta < 2 from tier two analyses using model averaging. All models within an AIC Delta < 2 warrant consideration along with the top performing model (Burnham et al. 2011; Burnham and Anderson 2002). We visually displayed data from nonaveraged models with stratified survival curves using the “surveminer” package in conjunction with “ggplot2” (Kassambara et al. 2021; Wickham 2016) following the methods of Denz and Timmesfeld (2023).

## 3 Results

Of 194 chicks hatched in the wild, 36 survived until fledging and 35 survived through the entire 13‐week monitoring duration, which exceeded the typical fledging period, which is estimated at around 12 weeks or 80–84 days. In total, we estimate that about 18.6% ± 2.8% (±se; 95% CI = 13.8%–24.9%) of chicks survived to fledging. Apparent survival probability was estimated at 68.6% ± 3.3% (±se; 95% CI = 62.3%–75.4%) from week one to two, at 53.6% ± 3.6% (±se; 95% CI = 47.0%–61.1%) from week two to three, and at 46.4% ± 3.6% (±se; 95% CI = 39.9%–54.0%) from week three to four (Table 1). Declines in apparent survival became more gradual and incremental after the first three survey periods, with no subsequent period having a point estimate for weekly mortality > 6% (Table 1).

**TABLE 1:** Estimated Whooping Crane chick survival probability (Prob. Surv.) across 12 survival periods (Period) spanning 13 weeks including estimated standard errors (SE), 95% confidence intervals (95% LCL and UCL), number of chicks remaining alive (# Chicks Alive) and mortality events (# Mort. Events) per age‐specific survey period in days (Days), weekly percent mortality (% Wk. Mort.), cumulative percent mortality (% Cumul. Mort.).

Period (weeks)	Days	# Chicks alive	# Mort. events	% Wk. Mort.	% Cumul. mort.	Prob. surv.	SE	95% LCL	95% UCL
1–2	7	194	61	31.4%	31.4%	68.6%	3.3%	62.3%	75.4%
2–3	14	133	29	14.9%	46.4%	53.6%	3.6%	47.0%	61.1%
3–4	21	104	14	7.2%	53.6%	46.4%	3.6%	39.9%	54.0%
4–5	28	90	11	5.7%	59.3%	40.7%	3.5%	34.4%	48.3%
5–6	35	79	10	5.2%	64.4%	35.6%	3.4%	29.4%	43.0%
6–7	42	69	11	5.7%	70.1%	29.9%	3.3%	24.1%	37.1%
7–8	49	58	7	3.6%	73.7%	26.3%	3.2%	20.8%	33.3%
8–9	56	51	7	3.6%	77.3%	22.7%	3.0%	17.5%	29.4%
9–10	63	44	3	1.5%	78.9%	21.1%	2.9%	16.1%	27.7%
10–11	70	41	1	0.5%	79.4%	20.6%	2.9%	15.6%	27.2%
11–12	77	40	4	2.1%	81.4%	18.6%	2.8%	13.8%	24.9%
12–13	84	36	1	0.5%	82.0%	18.0%	2.8%	12.6%	23.5%

Twelve models significantly outperformed their respective null models in predicting Whooping Crane weekly chick survival in the first phase of modeling efforts (Appendix 1). This included two models relating to *nests and chicks*, seven models related to *parental experience*, and three models related to *weather and climate covariates. Habitat and region* models did not significantly outperform their respective null models per a likelihood ratio test (Appendix 1). *Parental rearing history* models also did not outperform respective null models and demonstrated no significant impacts on chick survival. Nonetheless, the number of fledged chicks resulting from Whooping Cranes produced by more recent rearing methods (parent‐reared and wild‐hatched) remains limited.

**FIGURE 2:**
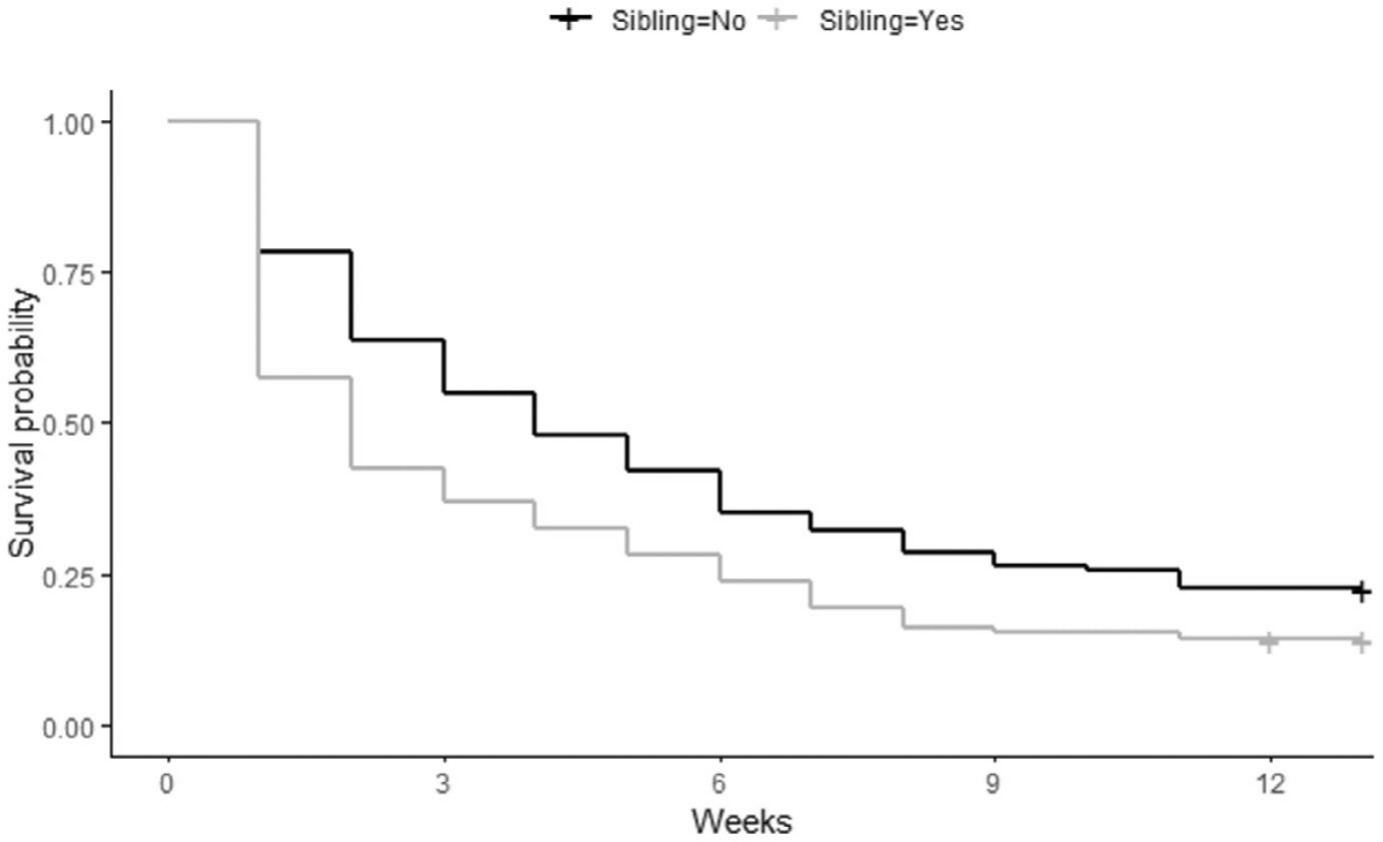
Whether or not a Whooping Crane chick had a sibling in relation to predicted chick survival probability across weekly survey periods during 2006–2023.

**FIGURE 3:**
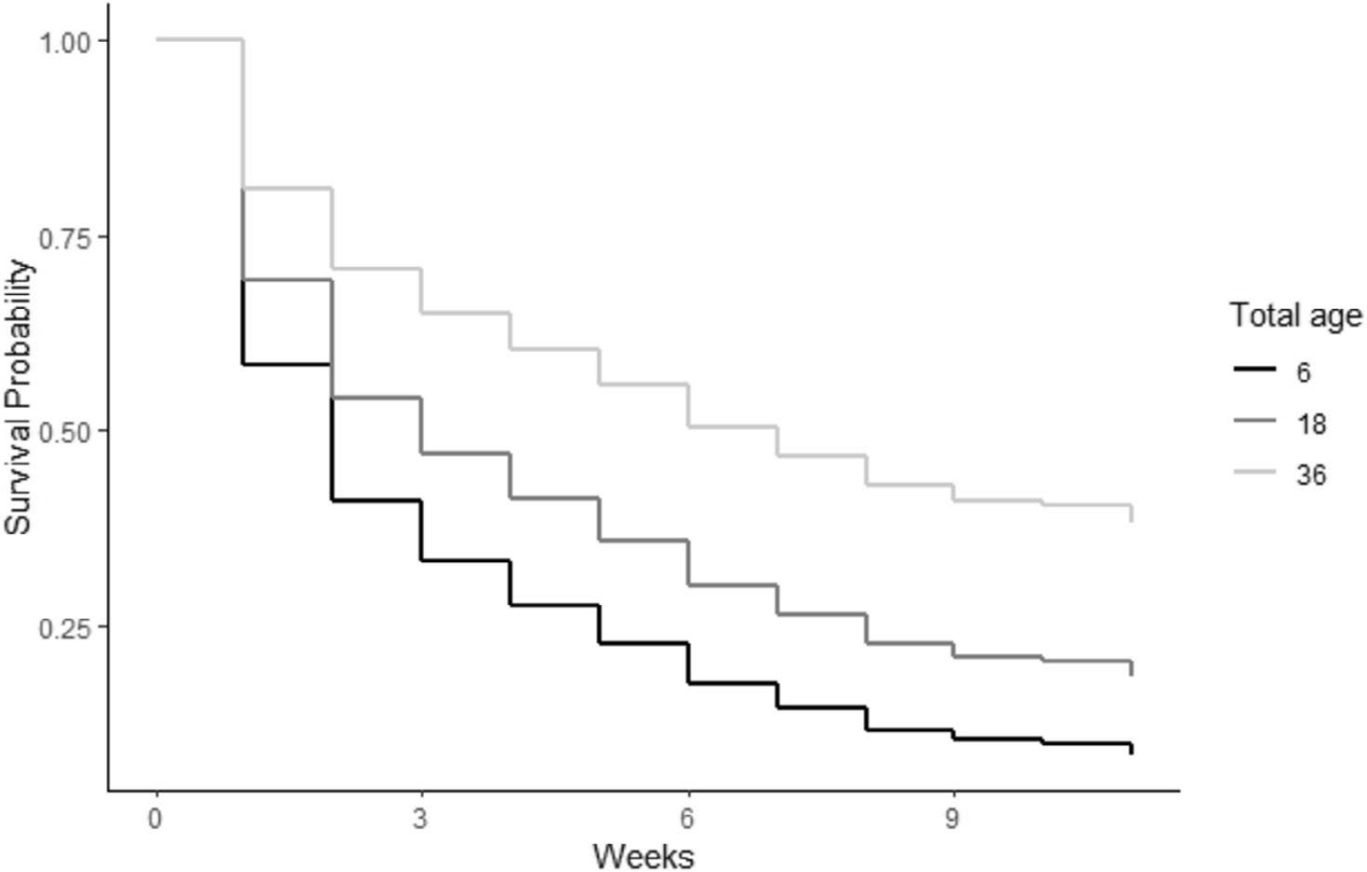
The combined age of nesting Whooping Crane pairs in years (Total age) in relation to predicted chick survival probability across weekly survey periods.

**FIGURE 4:**
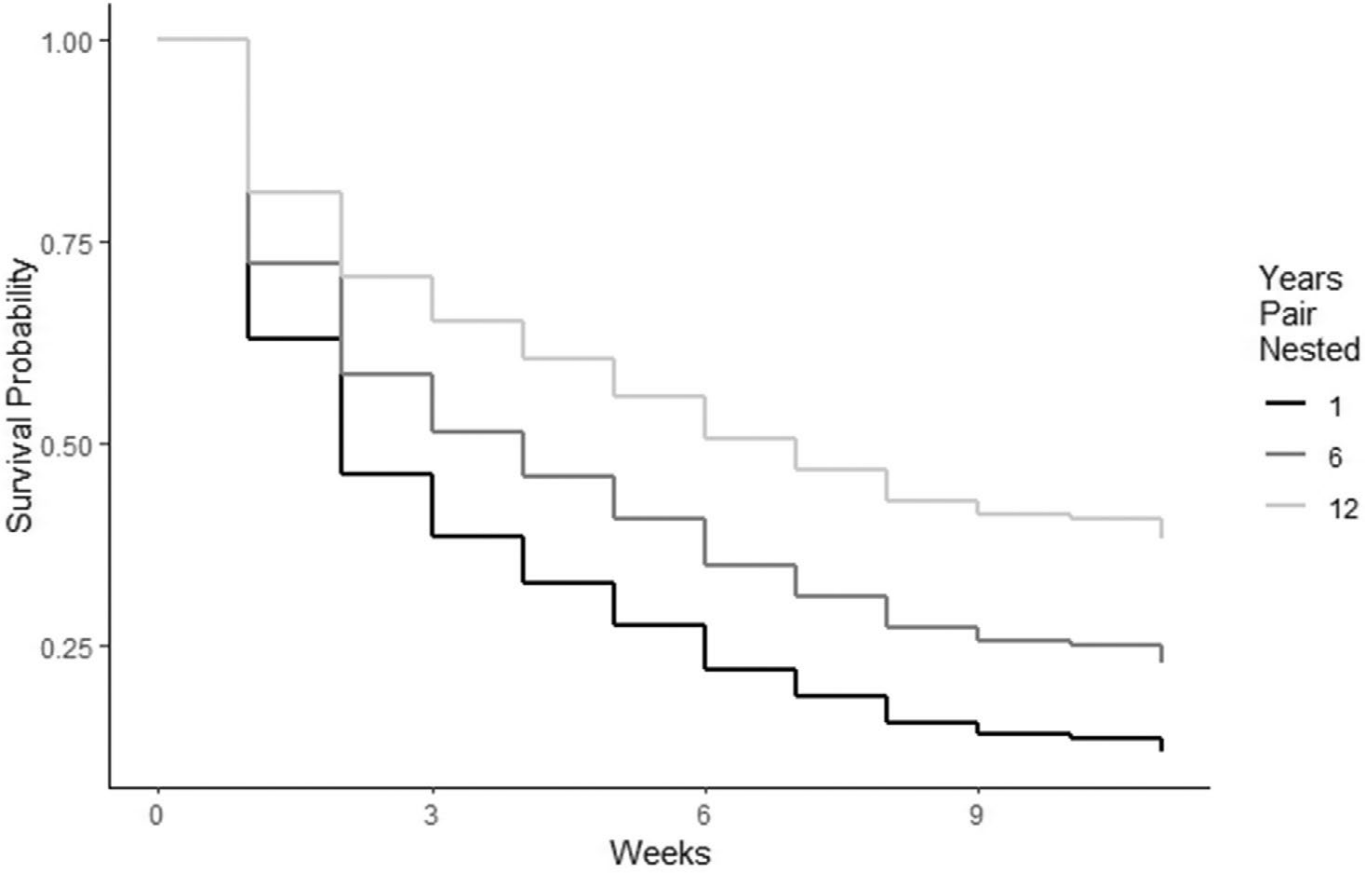
The total number of years that a Whooping Crane pair had nested together in relation to predicted chick survival probability across weekly survey periods during 2006–2023.

**FIGURE 5:**
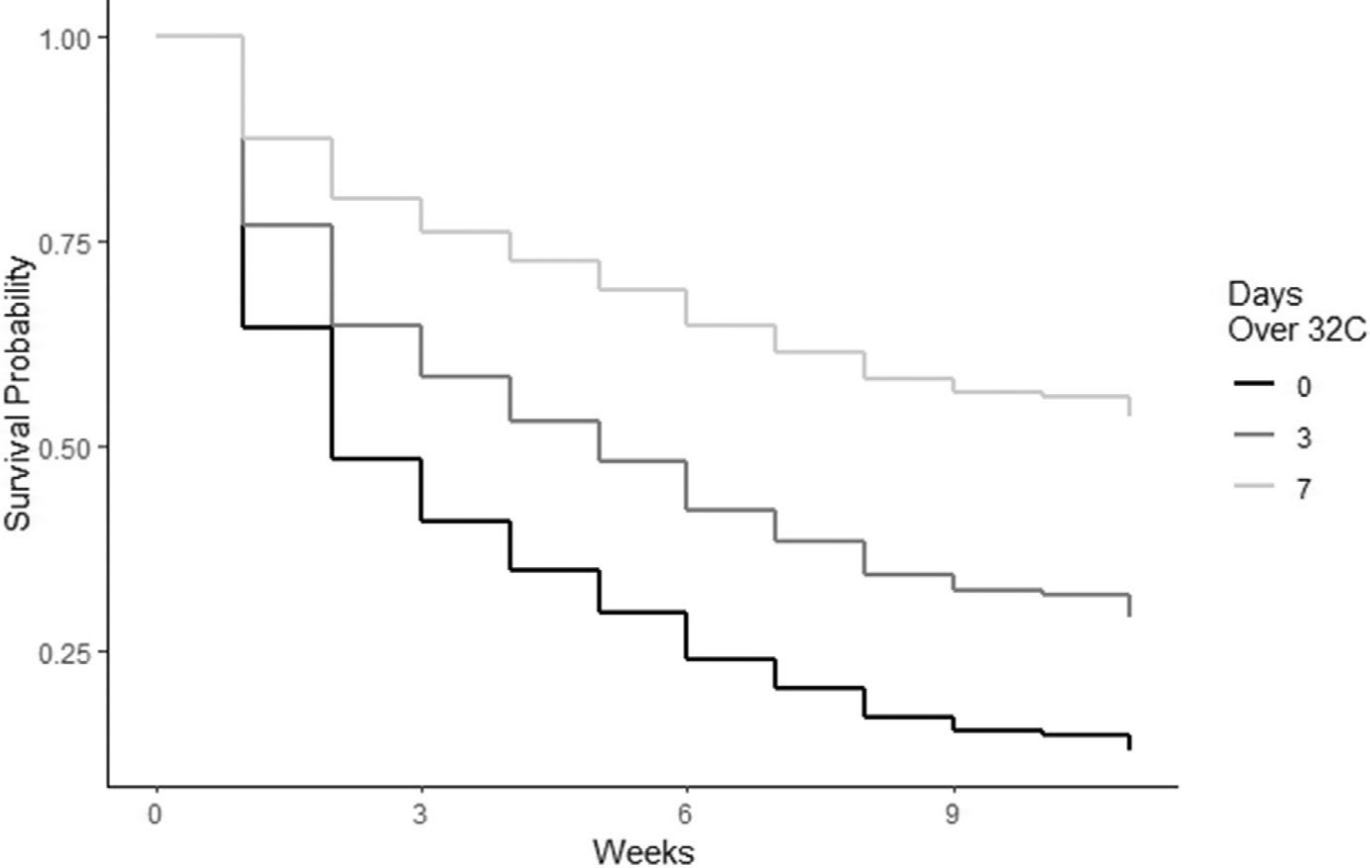
The total number of days that the maximum observed temperature exceeded 32°C (90°F) during the first 4 weeks of a Whooping Crane chick's life in relation to predicted chick survival probability across weekly survey periods during 2006–2023.

Pulling one egg from a two‐egg nest reduced the relative probability of chick mortality by 43.9% (95% CI = 0.9%–68.2%; *p* = 0.031). Similarly, if a chick had a sibling, its probability of prefledging mortality was increased by 49.7% (95% CI = 9.5%–104%; *p* = 0.012; Figure 2) relative to a chick without a sibling. The probability of prefledged chick mortality decreased by 4.9% (95% CI = −9.0% to −0.6%; *p* = 0.024) for each year of the female parent's age. Similarly, the probability of chick mortality decreased by about 5.2% for each year of the male parent's age (95% CI = −9.1% to −1.2%; *p* = 0.010). The combined age of the parents had a similar effect and reduced the probability of mortality by 3.1% for each unit increase (95% CI = −5.3% to −0.8%; *p* = 0.008; Figure 3). The number of years that the male parent (−5.7%; 95% CI = −9.8% to −1.5%; *p* = 0.007) and the female parent (−5.7%; 95% CI = −10.2% to −1.0%; *p* = 0.017) had each previously nested also reduced the probability of prefledged mortality for chicks. Concurrently, the number of years that the pair nested together decreased the relative probability of mortality by 7.0% per year (95% CI = −12.4% to −1.3%; *p* = 0.014; Figure 4). Finally, if the female parent previously fledged a chick, it also reduced the probability of mortality significantly (−29.7%; 95% CI = −50.5% to −0.2%; *p* = 0.043). The only other variables that outperformed the null model in predicting chick survival were various counts of the number of days during the chick development period when maximum daily temperatures exceeded 32°C (90°F). The best of these models suggested that with each additional day that exceeded 32°C during the first 4 weeks of life, the probability of chick mortality was reduced by 15.6% (95% CI = −23.8% to −6.6%; *p* = 0.0004; Figure 5).

Four models were within an AIC delta of 2 from tier‐two analyses that warranted consideration, including global model 4 as the top‐ranking model (Survival ~ Years Nesting Pair + Sibling + Days > 32 C in Weeks 1–4; Table 2). This also included global models 1, 2, and 5, which additionally incorporated “Years Nesting Male,” “Years Nesting Female,” and “Combined Age” (Table 2). Conditional model average results (parameter estimates are only averaged across models in which they are included) for all models within an AIC delta of 2 are presented in Table 3.

**TABLE 2:** Akaike information criterion corrected for sample size (AICc) model selection table for the tier‐2 modeling effort of Cox proportional hazard regression models including model name, variables, coefficient estimates, degrees of freedom, log likelihood estimation, AIC delta, and AIC weight (Wt.). For all models, *n* = 194, and the number of mortality events = 158.

Model	Days > 32 C Wks1–4	Sibling	Years nesting male	Combined Age	Male Age	Years nesting pair	Years nesting female	Female age	Prev. fledge female	Days > 32 C Wks1–9	Eggs pulled	Days > 32 C Wks1–13	df	AICc	Delta	Wt.
Global 4	−0.141	0.336	NA	NA	NA	−0.064	NA	NA	NA	NA	NA	NA	3	1456.8	0	0.233
Global 1	−0.132	0.315	−0.041	NA	NA	NA	NA	NA	NA	NA	NA	NA	3	1458.3	1.526	0.109
Global 5	−0.14	0.315	NA	NA	NA	NA	−0.044	NA	NA	NA	NA	NA	3	1458.3	1.526	0.109
Global 2	−0.138	0.291	NA	−0.021	NA	NA	NA	NA	NA	NA	NA	NA	3	1458.6	1.756	0.097
Global 7	−0.146	0.311	NA	NA	NA	NA	NA	NA	−0.272	NA	NA	NA	3	1458.8	2.008	0.085
Global 6	−0.142	0.309	NA	NA	NA	NA	NA	−0.036	NA	NA	NA	NA	3	1458.9	2.03	0.084
Global 9	−0.139	NA	NA	NA	NA	−0.064	NA	NA	NA	NA	−0.419	NA	3	1458.9	2.087	0.082
Global 3	−0.139	0.283	NA	NA	−0.032	NA	NA	NA	NA	NA	NA	NA	3	1459.3	2.481	0.067
Global 11	−0.137	NA	NA	−0.022	NA	NA	NA	NA	NA	NA	−0.332	NA	3	1460.4	3.621	0.038
Global 8	NA	0.340	NA	NA	NA	−0.074	NA	NA	NA	−0.051	NA	NA	3	1460.8	3.959	0.032
Days > 32 C Wks1–4	−0.17	NA	NA	NA	NA	NA	NA	NA	NA	NA	NA	NA	1	1460.9	4.053	0.031
Global 10	NA	0.266	NA	−0.029	NA	NA	NA	NA	NA	−0.054	NA	NA	3	1461.7	4.85	0.021
Yrs. Nesting male	NA	NA	−0.059	NA	NA	NA	NA	NA	NA	NA	NA	NA	1	1466.4	9.599	0.002
Combined age	NA	NA	NA	−0.031	NA	NA	NA	NA	NA	NA	NA	NA	1	1466.6	9.822	0.002
Days > 32 C Wks1–9	NA	NA	NA	NA	NA	NA	NA	NA	NA	−0.064	NA	NA	1	1466.9	10.078	0.002
Male age	NA	NA	NA	NA	−0.054	NA	NA	NA	NA	NA	NA	NA	1	1467.1	10.238	0.001
Sibling	NA	0.403	NA	NA	NA	NA	NA	NA	NA	NA	NA	NA	1	1467.3	10.456	0.001
Yrs. Nesting pair	NA	NA	NA	NA	NA	−0.072	NA	NA	NA	NA	NA	NA	1	1467.6	10.808	0.001
Yrs. Nesting female	NA	NA	NA	NA	NA	NA	−0.059	NA	NA	NA	NA	NA	1	1467.9	11.053	0.001
Days > 32 C Wks1–13	NA	NA	NA	NA	NA	NA	NA	NA	NA	NA	NA	−0.042	1	1468.1	11.233	0.001
Female age	NA	NA	NA	NA	NA	NA	NA	−0.05	NA	NA	NA	NA	1	1468.5	11.709	0.001
Eggs pulled	NA	NA	NA	NA	NA	NA	NA	NA	NA	NA	−0.578	NA	1	1469	12.136	0.001
Prev. Fledge female	NA	NA	NA	NA	NA	NA	NA	NA	−0.3524	NA	NA	NA	1	1469.5	12.724	0
Null	NA	NA	NA	NA	NA	NA	NA	NA	NA	NA	NA	NA	0	1471.6	14.775	0

**TABLE 3:** Conditional model average results for all variables in models of probability of ranking within an AIC delta of 2. This includes whether a Whooping Crane chick has a sibling, the number of years the pair has been nesting together, the number of days observed > 32°C (90°F) in the first 4 weeks of life, the number of years both the male and female have nested, and the combined age of the male and female parents. Exp(*B*) is the increase in the hazard ratio for a 1 unit change of the predictor variable. When *B* is negative, there is a decrease in the hazard ratio for a 1 unit change of the predictor variable.

Variable	*B*	SE	*Z*	*p*
Sibling—Yes	0.320	0.162	1.97	0.049
Years nesting pair	−0.064	0.031	2.05	0.041
Days > 32 C in Weeks 1–4	−0.138	0.053	2.59	0.010
Years nesting male	−0.041	0.024	1.67	0.095
Years nesting female	−0.044	0.027	1.67	0.095
Combined age	−0.021	0.013	1.61	0.107

Our top models suggest that the probability of survival to fledging for a chick without a sibling is 22.5%, whereas the probability for a chick with a sibling is estimated at 14.1%. A pair nesting together for the first time would have a 13.1% chance of success; this would increase to 21.8% after 6 years together, and to 34.6% after 12 years. The probability of fledging a chick when 0 days > 32°C are observed during the first 4 weeks of life is 13.7%. However, our models predict that this would be 26.5% when 3 days have exceeded 32°C in this period and 46.4% when more than 7 days exceeded this threshold early in the chick‐rearing season. A male parent that is in his first year of nesting was predicted by our models to have a 13.3% chance of fledging a chick. This increases to 21.2% in his eighth year, and 32.0% in his 16th year. Similarly, a female's chance of success is about 13.0% in her first year, but increases to 20.2% in year seven, and 31.8% in year 15. Nesting pair total age was a similarly useful predictor of chick survival probability, with only 12.0% predicted to fledge when the summed age was 6 years, but 18.5% predicted to fledge when the total age was 18 years, and a 30.7% chance when the summed age was 36 years.

## 4 Discussion

Whooping Crane population growth in the EMP is limited by low recruitment and elevated rates of chick mortality, as emphasized by our reported fledging rate (18.6% ± 2.8%). Our results are marginally elevated compared to fledging rates of EMP Whooping Cranes from 2010 to 2012 (16%; King et al. 2013a), noting that prior to 2010 only 1 Whooping Crane survived to fledge in the wild (WCEP 2007), an indication that fledging rates are slowly improving in the population. However, estimates of fledging rates in the self‐sustaining Aransas Wood Buffalo Population (36.4%; Bergeson et al. 2001) indicate as much as a 20.6% difference in fledging rates between populations and further support the relationship between high chick mortality and low recruitment in the EMP. Our study also found chick mortality was greatest following hatch and across the first 3 1‐week monitoring periods. Bergeson et al. (2001) and King et al. (2013a) reported elevated rates of Whooping Crane chick mortality in the first 7–22 days posthatch, which is consistent with our findings. Additionally, this observed relationship is not unique to Whooping Cranes, as many precocial and altricial chicks experience greater survival as they age due in part to an increase in experience, mobility, and size across the prefledging period (Fox et al. 2019; Maness and Anderson 2013; Schmidt 2022; Severud et al. 2022).

Another objective of our study was to identify factors affecting chick mortality or survival, and the results of our study indicate Whooping Crane chick mortality in the EMP is affected by parental experience, clutch size, and weather conditions, particularly early in the chick‐rearing period. In particular, the data from our study also suggest that greater parental experience may be the driving force behind chick survival and recruitment. King et al. (2013a) hypothesized chick fledge rates would increase in the EMP as the population ages and gains rearing experience. However, we have documented only a marginal increase. We believe this may be a result of Whooping Crane adults in the EMP also experiencing high rates of mortality due to predation, impact trauma primarily due to powerline collisions, gunshots, diseases, and more, leading to fewer experienced breeding adults in the population and fewer chicks surviving to fledge (Thompson et al. 2022b; Yaw et al. 2020). Management addressing adult and chick survival in tandem may be paramount to increased recruitment across the EMP. To date, studies have found no significant impact of captive rearing technique on chick survival postfledging (Thompson et al. 2022a) and rearing techniques of parents (captive vs. wild) were not found to be a significant predictor of wild‐hatched chick survival in this study. Perhaps additional observations related to more recently applied strategies will allow managers to develop a more refined understanding of the long‐term impacts of rearing history on parental efficacy to inform future management.

Clutch size also influenced chick survival. Specifically, chicks without siblings experienced a greater chance of survival. Our study supports nest management that reduces sibling competition for parental attention and resources as it has been found to positively impact the probability of chick survival and recruitment in the EMP. This finding should continue to influence our management of the population and indicates that there is likely limited harm and very likely even a benefit to the continued removal of eggs from the EMP for rearing in captivity. Current nest management in the EMP to increase population growth also involves removing whole clutches from first nests in areas disturbed by black fly emergence (Adler et al. 2019; Jaworski 2016; Thompson et al. 2022b) and may be used prior to single egg collection at second nests where applicable. Preliminary research suggests 64%–80% of re‐nesting attempts reach the hatching stage (Jaworski 2016); however, more research and a larger sample size are needed to determine re‐nesting propensity in the EMP following full‐clutch collection at first nests and the resulting impact on recruitment before it is considered for nest management outside of black fly disturbance control.

Warm weather, particularly early in the chick‐rearing period, was positively associated with fledging success. It is possible that these warm temperatures have an indirect impact on chick survival by effectively improving environmental conditions such as increasing food abundance or reducing the amount of time adults spend brooding, thus increasing the time spent foraging or provisioning. Other studies of birds have found similar results, particularly early in the rearing period (White Storks, *Ciconia ciconia* , Jovani and Tella 2004; Black‐throated Diver, *Gavia arctica* , Jackson 2005; Eurasian Coot, *Fulica atra* , Chyb and Minias 2022). Chyb and Minias (2022) documented contrasting effects of temperature on coot chick survival, where temperature was positively correlated with chick survival early in the rearing period and negatively correlated late in the period. Similarly, White Stork nestling survival was positively correlated with temperature. Storks were particularly sensitive to weather conditions prior to 20 days of age, and the authors suggested it could be due to a low homeothermy capacity of young birds (Jovani and Tella 2004). Jackson (2005) demonstrated that Black‐throated Diver chick survival was differentially impacted by temperatures depending on the predominant dietary resource exploited by parents. This highlights how temperature can regulate the prey base for chicks but that the impacts are context dependent. Whooping Cranes in the more northern breeding areas of the Aransas‐Wood Buffalo Population have higher fledging rates; however, there has not been a direct assessment of the effects of warm weather on fledging success in this population. For the EMP, given the density of nests and chicks at Necedah NWR, it is possible that the prey base at this particular site could be important to our results and warrant future exploration. Further investigation of the mechanism by which temperature affects Whooping Crane chick survival could inform reintroduction efforts or habitat management, especially as it relates to changes in climate and weather patterns.

Habitat variables were not significant drivers of chick survival in our study. However, other studies have found positive effects of habitat management on crane chick survival (e.g., growing season drawdowns of wetlands, McLean 2019). Therefore, further examination of how habitat characteristics may affect chick survival, predator detection, or food resources is warranted. The removal of woody vegetation is widely used as a habitat management tool to improve habitat for cranes throughout their life cycle and may affect nest success or chick survival (Dellinger et al. 2022; Lehnen et al. 2024; Pfeiffer and Currier 2005). To date, targeted control of woody species at EMP nesting sites has been limited, and future efforts to improve habitat conditions could benefit from applied research to evaluate success and facilitate adaptive management.

Whooping Crane chick survival is an important component of population growth for the EMP. Based on the results of this study, we recommend continuing current protocols to collect one egg from 2‐egg clutches for captive‐rearing as well as taking any actions to reduce adult mortality, so cranes live long enough to gain parental experience. Whooping Crane chicks without siblings had a higher probability of survival, so collecting one egg from 2‐egg clutches helps increase chick survival in the wild while also providing additional eggs for captive‐rearing and release into reintroduced populations. Further examination of how weather impacts crane chick survival also may inform future management of crane nesting territories or the surrounding habitat.

Long‐term conservation programs or reintroduction efforts benefit from research focused on improving techniques, evaluating management activities, and understanding limitations of the population in the wild (Seddon et al. 2007). Assessments of reproductive success, like this study, that include evaluations of management actions (e.g., egg collection), captive‐rearing techniques, behavior or experience, and site‐specific habitat are essential to the long‐term success of reintroduction efforts and can be applied to a wide variety of taxa and conservation programs.

## Author Contributions

**Hillary L. Thompson:** conceptualization (lead), data curation (lead), formal analysis (supporting), investigation (lead), methodology (equal), project administration (lead), visualization (supporting), writing – original draft (lead), writing – review and editing (lead). **Andrew J. Caven:** formal analysis (lead), methodology (equal), visualization (lead), writing – original draft (equal), writing – review and editing (supporting). **Stephanie M. Schmidt:** writing – original draft (equal). **Bianca R. F. Sicich:** data curation (equal), investigation (supporting), writing – original draft (supporting). **Alexis J. Sarrol:** data curation (equal), investigation (supporting), writing – original draft (supporting). **Eva K. Szyszkoski:** data curation (supporting), investigation (supporting), writing – review and editing (supporting). **Nicole M. Gordon:** data curation (supporting), investigation (supporting), writing – review and editing (supporting).

## Ethics Statement

This research was conducted in compliance with the *Ethical Guidelines for Statistical Practice*. No wild birds were handled specifically for this project.

## Conflicts of Interest

The authors declare no conflicts of interest.

## Data Availability

Our data are available on Dryad at: https://doi.org/10.5061/dryad.12jm63z8w.

## Appendix 1 Bivariate Cox Proportional Hazard Regression Models Including Model Theme, Model Number, Model Predictor Variable or Factor Levels, Data Type, Exponentiated Coefficient Displaying the Point Estimate for Survival Impacts, the 95% Confidence Intervals Surrounding the Point Estimate, the Variable or Factor‐Level's Significance via a *p* Value, the Sample Size Used for the Analysis, the Measure of Explained Variation Index, the Likelihood Ratio Chi‐Squared Test Result, and the *p* Value Indicating the Model's Significant Relative to the Null Model. Model and Variable Significance is Denoted as Follows: *p* < 0.01**, < 0.05*, < 0.10^, > 0.10^−^.

**Table:**

Theme	Mod. no.	Predictor variable	Type	exp(Coef)	95% LCL	95% UCL	Var. *p*	Var. sig.	*n*	MEV	Lik. ratio	Mod *p*	Mod. sig.
Nest and chick	1	Clutch size	Integer	1.086	0.636	1.854	0.763	—	175	0.00039	0.093	0.761	—
Nest and chick	2	Nest order	Integer	0.807	0.593	1.097	0.171	—	194	0.00740	1.927	0.165	—
Nest and chick	3	Eggs pulled—Yes	Factor	0.561	0.318	0.991	0.046	*	194	0.01800	4.664	0.031	*
Nest and chick	4	Hatch date	Numeric	0.996	0.986	1.005	0.378	—	194	0.00300	0.784	0.376	—
Nest and chick	5	Sibling—Yes	Factor	1.497	1.095	2.046	0.012	*	194	0.02400	6.344	0.012	*
Nest and chick	6	Sex—Male	Factor	0.508	0.108	2.394	0.392	—	34	0.05000	0.831	0.362	—
Parental experience	7	Female parent age	Integer	0.951	0.910	0.994	0.027	*	194	0.02000	5.092	0.024	*
Parental experience	8	Male parent age	Integer	0.948	0.909	0.988	0.012	*	194	0.02500	6.563	0.010	*
Parental experience	9	Combined age	Integer	0.969	0.947	0.992	0.010	**	194	0.02700	6.978	0.008	**
Parental experience	10	Years nesting female parent	Integer	0.943	0.898	0.990	0.019	*	194	0.02200	5.747	0.017	*
Parental experience	11	Years nesting male parent	Integer	0.943	0.902	0.986	0.009	**	194	0.02800	7.201	0.007	**
Parental experience	12	Years nesting pair	Integer	0.930	0.876	0.987	0.017	*	194	0.02300	5.992	0.014	*
Parental experience	13	Prev. hatch—1 parent	Factor	0.811	0.421	1.564	0.532	^	194	0.02100	5.593	0.061	^
Parental experience	13	Prev. hatch—2 parents	Factor	0.654	0.462	0.924	0.016	*	194	0.02100	5.593	0.061	^
Parental experience	14	Prev. fledge female parent—Yes	Factor	0.703	0.495	0.998	0.049	*	194	0.01600	4.077	0.043	*
Parental experience	15	Prev. fledge male parent—Yes	Factor	0.739	0.527	1.034	0.078	^	194	0.01200	3.217	0.073	^
Parental experience	16	Prev. fledge—1 parent	Factor	0.672	0.385	1.175	0.163	—	194	0.01800	4.797	0.091	^
Parental experience	16	Prev. fledge—2 parents	Factor	0.061	0.488	1.017	0.061	^	194	0.01800	4.797	0.091	^
Parental rearing	17	Female reared—Parent captive vs. Costume	Factor	1.157	0.666	2.012	0.604	—	194	0.00940	2.449	0.294	—
Parental rearing	17	Female reared—Wild vs. Costume	Factor	1.498	0.913	2.456	0.110	—	194	0.00940	2.449	0.294	—
Parental rearing	18	Male reared—Parent Captive vs. Costume	Factor	1.486	0.207	10.658	0.694	—	194	0.01500	3.895	0.143	—
Parental rearing	18	Male reared—Wild vs. Costume	Factor	2.818	1.140	6.966	0.025	*	194	0.01500	3.895	0.143	—
Parental rearing	19	Female released – Hard vs. Direct autumn release	Factor	0.925	0.492	1.741	0.809	—	194	0.01600	4.281	0.233	—
Parental rearing	19	Female released – Ultralight vs. Direct autumn release	Factor	0.750	0.499	1.126	0.165	—	194	0.01600	4.281	0.233	—
Parental rearing	19	Female released—Wild vs. Direct autumn release	Factor	1.198	0.670	2.142	0.543	—	194	0.01600	4.281	0.233	—
Parental rearing	20	Male released—Hard vs. Direct autumn release	Factor	1.414	0.187	10.712	0.737	—	194	0.01500	3.937	0.268	—
Parental rearing	20	Male released—Ultralight vs. Direct autumn release	Factor	0.947	0.564	1.590	0.836	—	194	0.01500	3.937	0.268	—
Parental rearing	20	Male released—Wild vs. Direct autumn release	Factor	2.683	0.970	7.422	0.057	^	194	0.01500	3.937	0.268	—
Habitat and region	21	Birthplace—Baraboo vs. Adams Co.	Factor	1.084	0.293	4.011	0.904	—	194	0.07600	20.142	0.126	—
Habitat and region	21	Birthplace—Colburn vs. Adams Co.	Factor	1.615	0.496	5.261	0.426	—	194	0.07600	20.142	0.126	—
Habitat and region	21	Birthplace—Grand River Marsh vs. Adams Co.	Factor	1.522	0.468	4.953	0.486	—	194	0.07600	20.142	0.126	—
Habitat and region	21	Birthplace—Green Lake Co. vs. Adams Co.	Factor	6.560	0.819	52.524	0.076	^	194	0.07600	20.142	0.126	—
Habitat and region	21	Birthplace—Horicon NWR vs. Adams Co.	Factor	1.013	0.339	3.027	0.981	—	194	0.07600	20.142	0.126	—
Habitat and region	21	Birthplace—Juneau Co vs. Adams Co.	Factor	0.708	0.273	1.837	0.478	—	194	0.07600	20.142	0.126	—
Habitat and region	21	Birthplace—McMillan SWA. vs. Adams Co.	Factor	6.560	0.819	52.524	0.076	^	194	0.07600	20.142	0.126	—
Habitat and region	21	Birthplace—Mead SWA vs. Adams Co.	Factor	1.632	0.628	4.241	0.315	—	194	0.07600	20.142	0.126	—
Habitat and region	21	Birthplace—Meadow Valley SWA vs. Adams Co.	Factor	1.607	0.494	5.227	0.431	—	194	0.07600	20.142	0.126	—
Habitat and region	21	Birthplace—Necedah NWR vs. Adams Co.	Factor	1.089	0.549	2.158	0.808	—	194	0.07600	20.142	0.126	—
Habitat and region	21	Birthplace—Portage Co. vs. Adams Co.	Factor	0.318	0.040	2.514	0.277	—	194	0.07600	20.142	0.126	—
Habitat and region	21	Birthplace—St. Croix Co. vs. Adams Co.	Factor	2.958	0.904	9.673	0.073	^	194	0.07600	20.142	0.126	—
Habitat and region	21	Birthplace—White River Marsh SWA. vs. Adams Co.	Factor	1.543	0.626	3.800	0.346	—	194	0.07600	20.142	0.126	—
Habitat and Region	21	Birthplace—Wood Co. vs. Adams Co.	Factor	0.278	0.060	1.287	0.102	—	194	0.07600	20.142	0.126	—
Habitat and Region	22	Region—Outside Necedah NWR vs. Necedah NWR	Factor	0.949	0.662	1.362	0.778	—	194	0.00130	0.348	0.840	—
Habitat and Region	22	Region—Rectangle vs. Necedah NWR	Factor	1.113	0.693	1.786	0.659	—	194	0.00130	0.348	0.840	—
Habitat and Region	23	Ecoregion—Central Sand Plains vs. Central Sandhills	Factor	0.984	0.500	1.938	0.963	—	194	0.02100	5.432	0.246	—
Habitat and Region	23	Ecoregion—Forest transition vs. Central Sandhills	Factor	1.685	0.667	4.257	0.270	—	194	0.02100	5.432	0.246	—
Habitat and Region	23	Ecoregion—Southeast Glacial Plains vs. Central Sandhills	Factor	1.265	0.553	2.891	0.578	—	194	0.02100	5.432	0.246	—
Habitat and Region	23	Ecoregion—Wester Prairie vs. Central Sandhills	Factor	2.752	0.840	9.016	0.095	^	194	0.02100	5.432	0.246	—
Habitat and Region	24	Protected land—Yes	Factor	1.295	0.882	1.902	0.187	—	194	0.00700	1.824	0.177	—
Habitat and Region	25	Nest site—Wetland size	Numeric	1.000	1.000	1.001	0.071	^	182	0.01000	2.595	0.107	—
Habitat and Region	26	Next site—Emergent wet meadow	Factor	0.755	0.467	1.221	0.252	—	188	0.00480	1.224	0.269	—
Habitat and Region	27	Nest site—Open water	Factor	0.975	0.674	1.412	0.895	—	188	0.00007	0.018	0.895	—
Habitat and Region	28	Nest site—Scrub shrub	Factor	1.096	0.739	1.625	0.649	—	188	0.00080	0.203	0.652	—
Habitat and Region	29	Nest site—Forest	Factor	0.749	0.367	1.526	0.426	—	188	0.00270	0.691	0.406	—
Habitat and Region	30	Winter region—North vs. Central	Factor	0.876	0.619	1.239	0.454	—	194	0.03000	7.777	0.051	^
Habitat and Region	30	Winter region—South vs. Central	Factor	0.813	0.448	1.474	0.495	—	194	0.03000	7.777	0.051	^
Habitat and Region	30	Winter region—Unknown vs. Central	Factor	0.259	0.080	0.833	0.023	*	194	0.03000	7.777	0.051	^
Weather and Climate	31	Yearly mean sunspot number	Numeric	1.002	0.998	1.006	0.284	—	194	0.00430	1.130	0.288	—
Weather and Climate	32	June PDSI	Numeric	1.023	0.956	1.094	0.517	—	194	0.00160	0.424	0.515	—
Weather and Climate	33	Total precipitation in Weeks 1–4	Numeric	1.046	0.960	1.140	0.301	—	194	0.00410	1.069	0.301	—
Weather and Climate	34	Total precipitation in Weeks 5–9	Numeric	0.997	0.923	1.076	0.932	—	194	0.00003	0.007	0.932	—
Weather and Climate	35	Total precipitation in Weeks 10–13	Numeric	0.992	0.924	1.064	0.815	—	194	0.00021	0.055	0.814	—
Weather and Climate	36	Total precipitation in Weeks 1–9	Numeric	1.013	0.963	1.067	0.611	—	194	0.00100	0.259	0.611	—
Weather and Climate	37	Total precipitation in Weeks 1–13	Numeric	1.004	0.965	1.045	0.827	—	194	0.00018	0.048	0.827	—
Weather and Climate	38	Maximum temperature across Weeks 1–4	Numeric	0.962	0.925	1.000	0.049	*	194	0.01400	3.720	0.054	^
Weather and Climate	39	Maximum temperature across Weeks 5–9	Numeric	0.958	0.907	1.013	0.132	—	194	0.00900	2.337	0.126	—
Weather and Climate	40	Maximum temperature across Weeks 10–13	Numeric	0.992	0.956	1.029	0.659	—	194	0.00075	0.195	0.659	—
Weather and Climate	41	Average temperature across Weeks 1–4	Numeric	0.979	0.950	1.008	0.158	—	194	0.00760	1.965	0.161	—
Weather and Climate	42	Average temperature across Weeks 5–9	Numeric	1.001	0.924	1.085	0.979	—	194	0.00000	0.000	0.979	—
Weather and Climate	43	Average temperature across Weeks 10–13	Numeric	1.006	0.959	1.057	0.799	—	194	0.00025	0.066	0.798	—
Weather and Climate	44	Minimum temperature in Weeks 1–4	Numeric	0.991	0.972	1.009	0.321	—	194	0.00380	0.985	0.321	—
Weather and Climate	45	Minimum temperature in Weeks 5–9	Numeric	1.022	0.986	1.058	0.237	—	194	0.00550	1.426	0.233	—
Weather and Climate	46	Minimum temperature in Weeks 10–13	Numeric	1.013	0.983	1.043	0.399	—	194	0.00280	0.730	0.393	—
Weather and Climate	47	Count of days of > 32 C in Weeks 1–4	Integer	0.844	0.762	0.935	0.001	**	194	0.04900	12.747	0.000	***
Weather and Climate	48	Count of days of > 32 C in Weeks 5–9	Integer	0.972	0.910	1.037	0.386	—	194	0.00300	0.783	0.376	—
Weather and Climate	49	Count of days of > 32 C in Weeks 10–13	Integer	0.962	0.892	1.037	0.312	—	194	0.00430	1.118	0.290	—
Weather and Climate	50	Count of days of > 32 C in Weeks 1–9	Integer	0.938	0.892	0.987	0.013	*	194	0.02600	6.723	0.010	**
Weather and Climate	51	Count of days of > 32 C in Weeks 1–13	Integer	0.959	0.924	0.995	0.025	*	194	0.02100	5.568	0.018	*
Weather and Climate	52	Count of days of < 0 C in Weeks 1–4	Integer	0.989	0.835	1.172	0.900	—	194	0.00006	0.016	0.899	—
Weather and Climate	53	Count of days of < 0 C in Weeks 1–13	Integer	0.951	0.797	1.136	0.580	—	194	0.00120	0.319	0.572	—
